# Supplementary material for: Reconstructing noisy gene regulation dynamics using extrinsic-noise-driven neural stochastic differential equations
Source: PLoS Comput Biol. 2025 Sep 17;21(9):e1013462. doi: 10.1371/journal.pcbi.1013462 (PMC12513633; doi:10.1371/journal.pcbi.1013462)
Supplement: S1 Text — (PDF) [file pcbi.1013462.s001.pdf]

S1 Text: Technical Appendices

Appendix A: Hyperparameters and conditions used in the neural SDE model

In this section, we provide the hyperparameters in the neural SDE models as well as the training details in Table A in S1 Text.

Table A: The hyperparameters for training the neural SDE model of each example.

	Example 1	Example 2	Example 3
Gradient descent method	Adam	Adam	Adam
Learning rate	0.002	0.002	0.002
Weight decay	0.005	0.005	0.005
Activation function	ReLU	ReLU	ReLU
# of epochs	500	2000	2000
# of hidden layers in $\hat{f}$	3	2	2
# of neurons in hidden layer in $\hat{f}$	100	200	400
# of hidden layers in $\hat{\sigma}$	3	2	2
# of neurons in each hidden layer in $\hat{\sigma}$	100	200	400
Δt	0.1 (s)	$\frac{1}{720}$ (hr)	$\frac{1}{12}$ (hr)
T	11	361	31

Appendix B: RPA dynamic binding to long ssDNA can be simulated by a generalized random sequential adsorption (RSA) model

To elucidate the biophysical mechanism of replication protein A (RPA) binding dynamics to long single-stranded DNA (ssDNA), we developed a continuous-time discrete Markov chain model. The Random Sequential Adsorption (RSA) model in a one-dimensional (1D) finite-length context effectively represents the process of protein binding to DNA, capturing the key property that each nucleotide (nt) of ssDNA cannot be occupied by more than one protein molecule. This unique characteristic leads to incomplete occupation even with protein oversaturation, distinguishing DNA-relevant reactions from those described by the mass action law. To reveal finer structures such as gap distribution, we implemented an exact stochastic sampling approach.

We adapted the model based on current knowledge of RPA binding modes, incorporating multiple binding modes and volume exclusion effects. RPA has two binding modes: a 20-nt mode (partial binding mode, PBM) and a 30-nt mode (full-length binding mode, FLBM). RPA initially binds to a 20-nt ssDNA with a rate of k_1 and dissociates at a rate of k_{-1}. This 20-nt mode assumes constant k_1. One scenario involves DBD-A, DBD-B, and DBD-C binding to ssDNA, with subsequent DBD-D binding leading to the 30-nt mode. In the 20-nt mode, DBD-D binds an extra 10-nt ssDNA with a rate of k_2 and dissociates at k_{-2}, forming the 30-nt mode (FLBM). RPA always aligns in the same direction along DNA.

The multiple binding modes result in interesting kinetic features like facilitated exchange and desorption. In this model, the ssDNA fragment state is represented by a vector of length L with each component

taking values in $\{0, 1\}$, where 0 indicates an unoccupied nucleotide and 1 indicates an occupied one. Each RPA initially occupies $\ell = 20$ nts and can further occupy $\Delta\ell = 10$ nts if the local state allows, moving in the 3' direction. Each DNA site can be occupied by only one RPA molecule, altering the RSA model's available reactions. Each consecutive unoccupied segment of length ℓ recruits one RPA at the rate of k_1, with the total binding rate given by:

$$v_1 = k_1 \sum_{j=1}^{L-\ell+1} \delta_{\mathbf{0}^{\times \ell}}(\text{state}[j, j+1, \ldots, j+\ell-1]),$$

where $\delta_a(b)$ is the Kronecker delta function, $\mathbf{0}^{\times \ell}$ is a zero vector of length ℓ, and state$[j, \ldots, j+\ell-1]$ is the ssDNA state vector.

To occupy another 10 nts, we assigned a rate parameter k_2 and calculate the overall rate by:

$$v_2 = k_2 \sum_{q_j} \delta_{\mathbf{0}^{\times \Delta\ell}} \left(\text{state}[q_j + \ell, \ldots, q_j + \ell + \Delta\ell - 1]\right),$$

where q_j represents the leftmost position of each bound RPA in the 20-nt mode. For unbinding, the 30-nt mode reopens to the 20-nt mode at rate k_{-2}, and the 20-nt mode desorbs at rate k_{-1}. The overall rates are:

$$v_{-2} = k_{-2}\#\{\text{FLBM RPA}\},$$
$$v_{-1} = k_{-1}\#\{\text{PBM RPA}\}.$$

The total possible reaction rate is:

$$v_{\text{tot}} = v_1 + v_{-1} + v_2 + v_{-2}.$$

Reactions occur stochastically according to exponentially distributed waiting times with parameter v_{tot}. The waiting time δt follows the exponential distribution with a probability density function:

$$\text{pdf}(\delta t = t) = v_{\text{tot}} e^{-v_{\text{tot}} t}.$$

After each reaction, the DNA state updates, and the possible reactions are re-evaluated. The Gillespie algorithm was used to sample the trajectories of this stochastic model. We used Julia to perform exact stochastic simulations of all known RPA-ssDNA interactions, and codes are available at (`https://github.com/hsianktin/RPA_model`).

Appendix C: Implementation of benchmarks

In this section, we introduce the benchmark methods; hyperparameters and settings for training of all methods used in this paper are shown in Table B in S1 Text.

RNN networks

Recurrent neural networks (RNN) are often used for language processing, but they can also be used to analyze time series data with temporal correlations. For reconstructing the RPA-DNA binding dynamics, we used the RNN model. A neural network that contains two layers of RNN and two linear layers was used to model RPA's dynamic binding with single-stranded DNA. All layers of RNN are built using `torch.nn.Module` package. Hyperparameters in the neural network are initialized by Pytorch default settings. The parameters of this neural network are initialized by default. At each time step, $\boldsymbol{X}(t; \omega) \in \mathbb{R}^d$ representing the RPA dynamics at this time point is inputted in Example 2.2.2, and the dynamics at the next time point: $\boldsymbol{X}(t + \Delta t; \omega)$ is outputted as the prediction. The RNN is trained by optimizing the loss function Eq. (8). The pipeline of the model could be seen in Fig A in S1 Text.

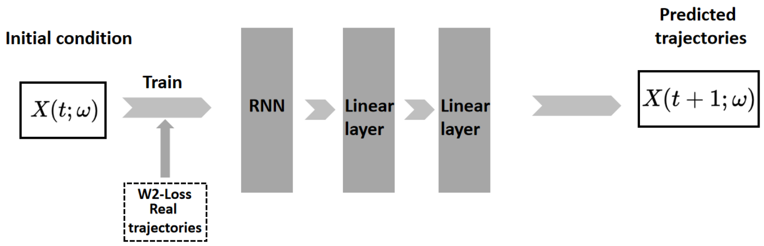

Figure A: The structure of the RNN model used. The RNN layer in this figure can be built using `torch.nn.RNN`.

LSTM networks

Long Short-Term Memory (LSTM) networks , a class of variants of Recurrent Neural Networks (RNNs), have been widely used in modeling sequential data such as time series and natural language. We used the LSTM network model implemented through the `torch.nn.Module` package for reconstructing the RPA-DNA dynamics. Hyperparameters in the neural network are initialized by default. At each time step, the $\boldsymbol{X}(t;\omega) \in \mathbb{R}^d$ discussed above is inputted in Example 2.2.2, and the state at the next time point: $\boldsymbol{X}(t+\Delta t;\omega)$ is outputted as the prediction. Then, the gradients of the loss function Eq. (8) are calculated to update the parameters in the LSTM model. The input size of the two layers of LSTM is 2 and the hidden size is 4. The input size and output size of the two linear layers are 8,4 and 4,2 respectively. The LSTM is trained by optimizing the loss function Eq. (8). The pipeline of the model is shown in Fig B in S1 Text.

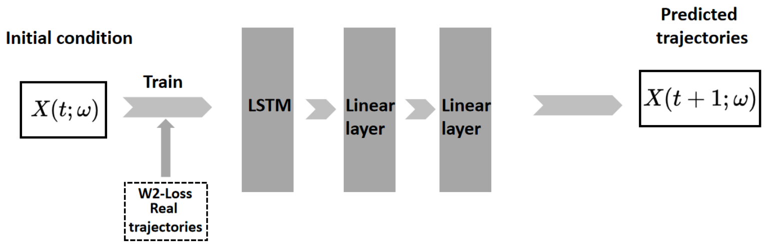

Figure B: The structure of the LSTM model used. The LSTM layer in this figure can be built using `torch.nn.LSTM`.

Gaussian Process

The Gaussian Process (GP) is also widely used in modeling time-series data; it is implemented using the `gaussianprocess.GaussianProcessRegressor` package in Python. We used radial basis functions as kernel functions (using sklearn.gaussianprocess.kernels package), and the hyperparameters of α and n-restarts-optimizer are 0.3 and 5, respectively. When training the GP model, we inputted the trajectories of all time points in the training set to the GP for fitting. Then, we inputted the trajectory of the current time point in the testing set, and the model predicts the trajectory of the next time point based on the kernel.

Appendix D: Generating the simulated NFκB dataset

To evaluate the performance of our proposed END-nSDE method in reconstructing dynamics of NFκB signaling, we generated training trajectories by simulating a previously developed 52-dimensional model

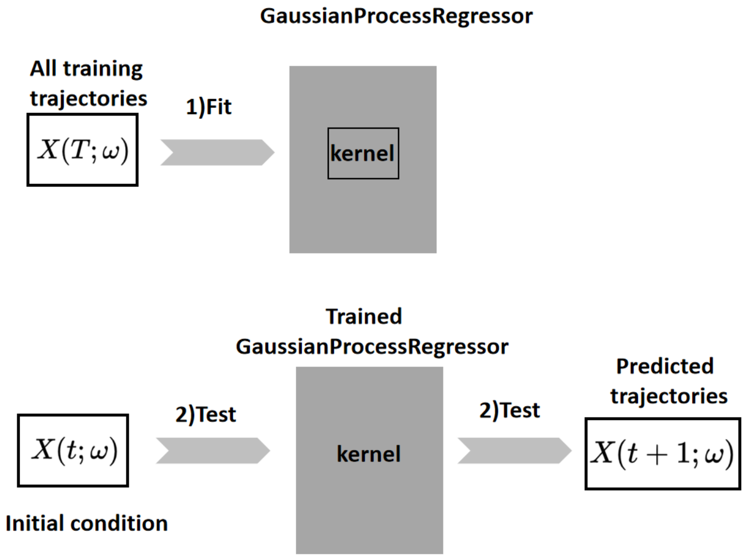

Figure C: The structure of the GP model used in this work.

Table B: Hyperparameters of benchmark methods

Model	Implementation
RNN	one RNN layer plus four MLP layers
	width of RNN layer: 256
	learning rate: 5×10^{-4}
	learning epoch: 2000
	Optimization method: Adam
LSTM	one LSTM layer plus four MLP layers
	width of LSTM layer: 128
	learning rate: 5×10^{-4}
	learning epoch: 3000
	Optimization method: Adam

for the NFκB signaling network under 100ng/mL TNF stimulation (see Eqs. (10) for the SDEs). The synthetic trajectories were generated using 100 sets of noise intensities. We set $\sigma_1 \in [10^{-3.2}, 10^{-2.2}], \sigma_2 \in [10^{-2.5}, 10^{-1.5}]$ in Eqs. (10) and use 100 combinations of (σ_1, σ_2): $\sigma_1 \in \{10^{-3.2+i\delta_1}, i = 0, \ldots, 9\}, \delta_1 = 0.1$ and $\sigma_2 \in \{10^{-2.5+j\delta_2}, j = 0, \ldots, 9\}, \delta_2 = 0.1$ respectively. Other parameters were fixed constants. The parameter values in Eqs. (10) are listed in Table C in S1 Text.

The corresponding SDEs were simulated using the 'SDEProblem' function from the 'DifferentialEquations' package in Julia. Simulations were conducted from 0 minutes (stimulus application time) to 150 minutes, and the state was recorded at every 5 minute intervals. Initial values were set to the steady-state solutions of the ordinary differential equations (ODEs), which were obtained using the ode15s function in MATLAB.

Table C: Parameter values for the NFκB model.

Parameter	Value	description
k_{basal}	5×10^{-7}	basal IκBα mRNA synthesis
$k_{\max}$	6×10^{-5}	maximal rate of IκBα mRNA synthesis induced by NFκB
$n_{\text{NF}\kappa\text{B}}$	2.938	Hill coefficient for mRNA syn
$K_{\text{NF}\kappa\text{B}}$	0.1775	EC50 for mRNA syn
k_{deg}	0.33	degradation rate of IκBα mRNA
k_{imp}	0.6	import rate of NFκB
$k_{\text{a-I}\kappa\text{B-NF}\kappa\text{B}}$	200	association rate of NFκB and IκBα
$k_{\text{deg-NF}\kappa\text{B}}$	0	degradation rate of NFκB
k_{exp}	0.042	export rate of NFκB
v	3.5	Volume ratio: cytoplasmic volume/nuclear volume
$k_{\text{d-I}\kappa\text{B-NF}\kappa\text{B}}$	0.008	dissociation rate of NFκB-IκBα
k_{phos}	2	Phosphorylation/degradation of complexed IκBα

Appendix E: Training a neural SDE using simulated NFκB trajectories

We partitioned the simulated ground-truth trajectories into training and validation sets as follows: 50% of each of 96 sets of trajectories (out of a total of 121) associated with different noise intensities were used for training, while the remaining simulated ground-truth trajectories were used as the validation dataset. Since we could only observe NFκB activity, we defined our loss function (see Eq. (8)) to focus solely on the nuclear NFκB and the IκBα-NFκB nuclear complex. In the loss function Eq. (8), $\omega \equiv (\sigma_1, \sigma_2)$ denoted two noise intensities (see Eqs. (10)). $\mu(\omega)$ and $\hat{\mu}(\omega)$ are the distributions of $\boldsymbol{X}(t;\omega)$ and $\hat{\boldsymbol{X}}(t;\omega)$, respectively. $\boldsymbol{X}(t;\omega)$ represented the values of (u_5, u_{10}) in Eqs. (10) at time t. The other 50 variables were not included in the calculation of the loss function.

Appendix F: Reconstructing IκBαNFκBn and NFκBn in NFκB signaling as a 2D SDE model

To investigate the importance of latent variables that are not included in the loss function, instead of reconstructing the 52D surrogate SDE model for the NFκB signaling dynamics, we attempted to directly reconstruct the dynamics of the nuclear complex IκBα−NFκB and nuclear NFκB using the 2D SDE

$$\begin{aligned} \mathrm{d}\hat{u}_5 &= f_5(\hat{u}_5, \hat{u}_{10}, t)\mathrm{d}t + \sigma_1(\hat{u}_5, \hat{u}_{10}, t)\mathrm{d}B_{1,t}, \\ \mathrm{d}\hat{u}_{10} &= f_{10}(\hat{u}_5, \hat{u}_{10}, t)\mathrm{d}t + \sigma_2(\hat{u}_5, \hat{u}_{10}, t)\mathrm{d}B_{2,t} \end{aligned} \tag{1}$$

to approximate the two SDEs in NFκB signaling model that describes nuclear IκBα-NFκB complex and NFκB.

Using a 2D SDE model to reconstruct the NFκB signaling dynamics could not accurately reconstruct the noisy dynamics of nuclear IκBα-NFκB and NFκB. As shown in Fig D G-H, for certain noise strengths (σ_0, σ_1), both the training and testing losses (the temporally decoupled squared W_2 distance Eq. (3)) are large compared to those obtained from the direct reconstruction of the full 52-dimensional model. Furthermore, when providing the inferred noise strengths from experimentally observed trajectories (the same as in Fig 7A and 7B), the reconstructed 2D SDE fails to generate predicted trajectories $(\hat{u}_5, \hat{u}_{10})$ that align well with experimental data. Thus, it is necessary to retain the remaining 50 variables in the model, although they are not directly used in the calculation of the loss function.

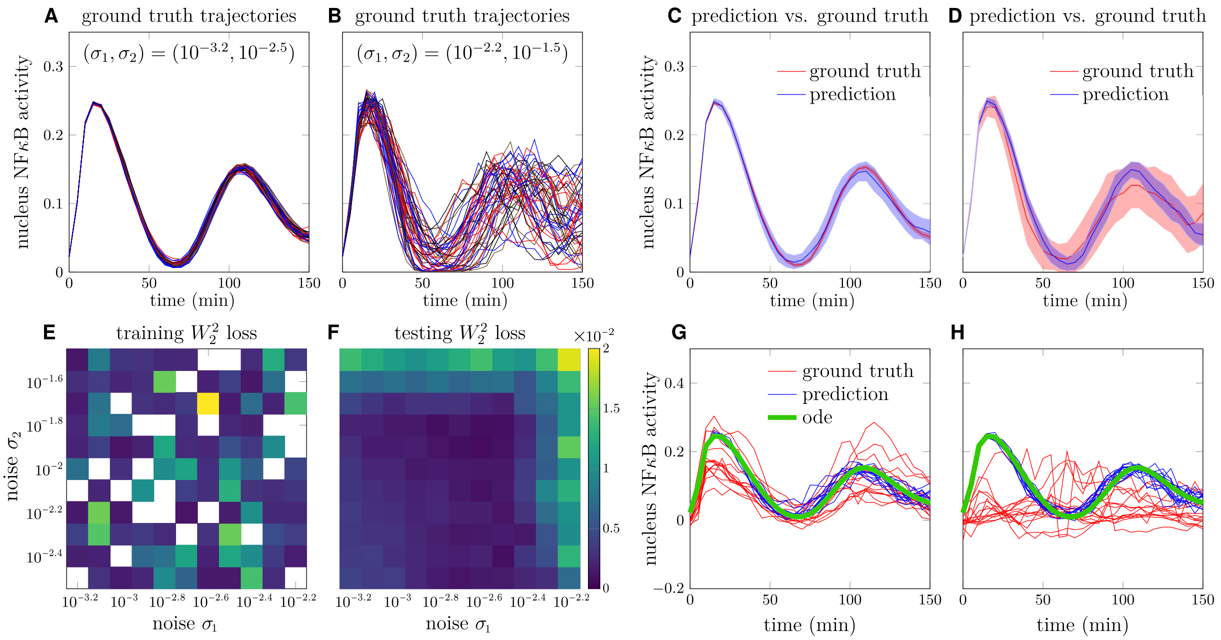

Figure D: **Reconstructed NFκB dynamics using a two-dimensional nSDE.** A. Trajectories of nuclear NFκB concentration over time in the synthetic dataset with noise intensities $\sigma_1 = 10^{-3.2}$, $\sigma_2 = 10^{-2.5}$. B. trajectories of nuclear NFκB concentration over time in the synthetic dataset with noise intensities $\sigma_1 = 10^{-2.2}$, $\sigma_2 = 10^{-1.5}$. C. Comparison of NFκBn trajectories predicted by the neural SDE with the ground truth trajectories under noise intensities $\sigma_1 = 10^{-3.2}$, $\sigma_2 = 10^{-2.5}$. D. Comparison of NFκBn predicted by the neural SDE with the ground truth trajectories under noise intensities $\sigma_1 = 10^{-2.2}$, $\sigma_2 = 10^{-1.5}$. E. The squared W_2 distance between distributions of predicted trajectories and ground truth trajectories in the training set under different noise strengths σ_0, σ_1. Empty cells indicate that the corresponding parameter set is not included in the training set. F. The squared W_2 distance between distributions of the predicted trajectories and the ground truth trajectories on the testing set. G.-H. After grouping the experimental trajectories by cosine similarity to the ODE reference trajectory (shown in green), the trained neural network estimates the noise (σ_1, σ_2) of each group of experimental trajectories (shown in red). Then, these estimates were fed into the nSDE to generate a group of (reconstructed) trajectories (shown in blue). The highest- and lowest-ranked similarity groups (#1 and #31, see Fig 9D, 9E, and 9I) are shown in G. and H., respectively.

Appendix G: Dividing experimental NFκB trajectories into subgroups

We divided the experimentally observed trajectories into 32 groups, each consisting of 32 trajectories. The experimental data were divided into different groups based on each trajectory's correlation with a ODE-model-based deterministic trajectory

$$\mathrm{corr}(\mathbf{v}_j, \mathbf{v}_{\mathrm{ODE}}) = \frac{\sum_{i=1}^{n} v_j(t_i) v_{\mathrm{ODE}}(t_i)}{\sqrt{\sum_{i=1}^{n} v_j^2(t_i)} \cdot \sqrt{\sum_{i=1}^{n} v_{\mathrm{ODE}}^2(t_i)}} \tag{2}$$

where $\mathbf{v}_j(t_i), \mathbf{v}_{\mathrm{ODE}}(t_i)$ denote the jth observed trajectory in experimental data and the ODE trajectory. The closer a trajectory is to the first-principle-based ODE trajectories, the higher probability that the fluctuations result from intrinsic noise (*i.e.* the Brownian-type noise).

Appendix H: Training the neural network to infer noise intensities in NFκB dynamics

We adopted a neural network that takes a group of trajectories $(u_5(t) + u_{10}(t))$ under the same noise intensity (σ_1, σ_2) as the input and outputs the inferred noise intensity $(\hat{\sigma}_1, \hat{\sigma}_2)$. Weights and biases in this neural network were optimized by minimizing the mean squared error (MSE) loss:

$$\mathrm{MSE}(\Lambda) = \sum_{i=1}^{m} \|\sigma_i - \hat{\sigma}_i\|^2, \tag{3}$$

where σ_i are the ground truth noise intensity parameters underlying the ith group of observed trajectories and $\hat{\sigma}_i$ are the corresponding predicted parameters. Despite the assumption of all cells sharing the same drift function (the same underlying dynamics), different trajectories naturally arise from intrinsic noise.

Detailed steps on inferring noise intensities from a group of trajectories are provided in Fig EA, EB, and EC. The training and testing sets are the same as those in Appendices and of the Supporting Information. The workflow for splitting NFκB SDE-simulated trajectories into training and testing datasets is illustrated in Fig EA and EB. Specifically, out of the 121 combinations of noise intensities (each containing 100 simulation trajectories), 20% were designated for the testing dataset pool. For the remaining 80% noise intensities, 50% of the trajectories under each parameter were randomly selected and added to the training dataset pool (indicated by the olive box in Fig EB). The remaining 50% of trajectories from these parameter sets were used for the testing dataset pool (red box in Fig EB). From both the training and testing dataset pools, for each combination of noise intensity, a group of trajectories was randomly sampled using a permutation sampling approach to construct the training and testing datasets (olive and red solid box in Fig EB).

The neural network used is equipped with an attention structure followed by a feed-forward structure of 2 hidden layers with 64 and 128 neurons in each layer, respectively, where the attention mechanism is designed for assigning weights to different trajectories in a group (sequenced by their similarities to the deterministic ODE trajectory) (Fig EC). The hidden dimension of the attention structure in the query layer is 30, and the hidden dimension of the attention structure in the key and value layers is both 32.

To assess the impact of group size on the performance of the trained neural network in predicting noise intensities, we tested group sizes corresponding to 2, 4, 8, 16, and 32 trajectories per group. The accuracy of the predictions was evaluated using the relative error metric:

$$\frac{|\lg(\hat{\sigma}_1) - \lg(\sigma_1)| + |\lg(\hat{\sigma}_2) - \lg(\sigma_2)|}{|\lg(\sigma_1)| + |\lg(\sigma_2)|}.$$

To explore how the proposed noise inference depends on the frequency of trajectory sampling, we trained the neural network (Fig EC.) using different numbers of sampled time points. Fig F plots the errors with

downsampled time points, plotted in terms of the time interval $\Delta t = 150min/\big(\#\,\text{time points} - 1\big)$, and shows that the error in the predicted noise intensity is fairly insensitive to reducing the number of time points, down to about 6 time points (30 minute intervals). By using a group of 32 trajectories, inference of the intrinsic noise intensity, allows a smaller number of observed time points. In our example, errors increase appreciably when fewer than three time points are used.

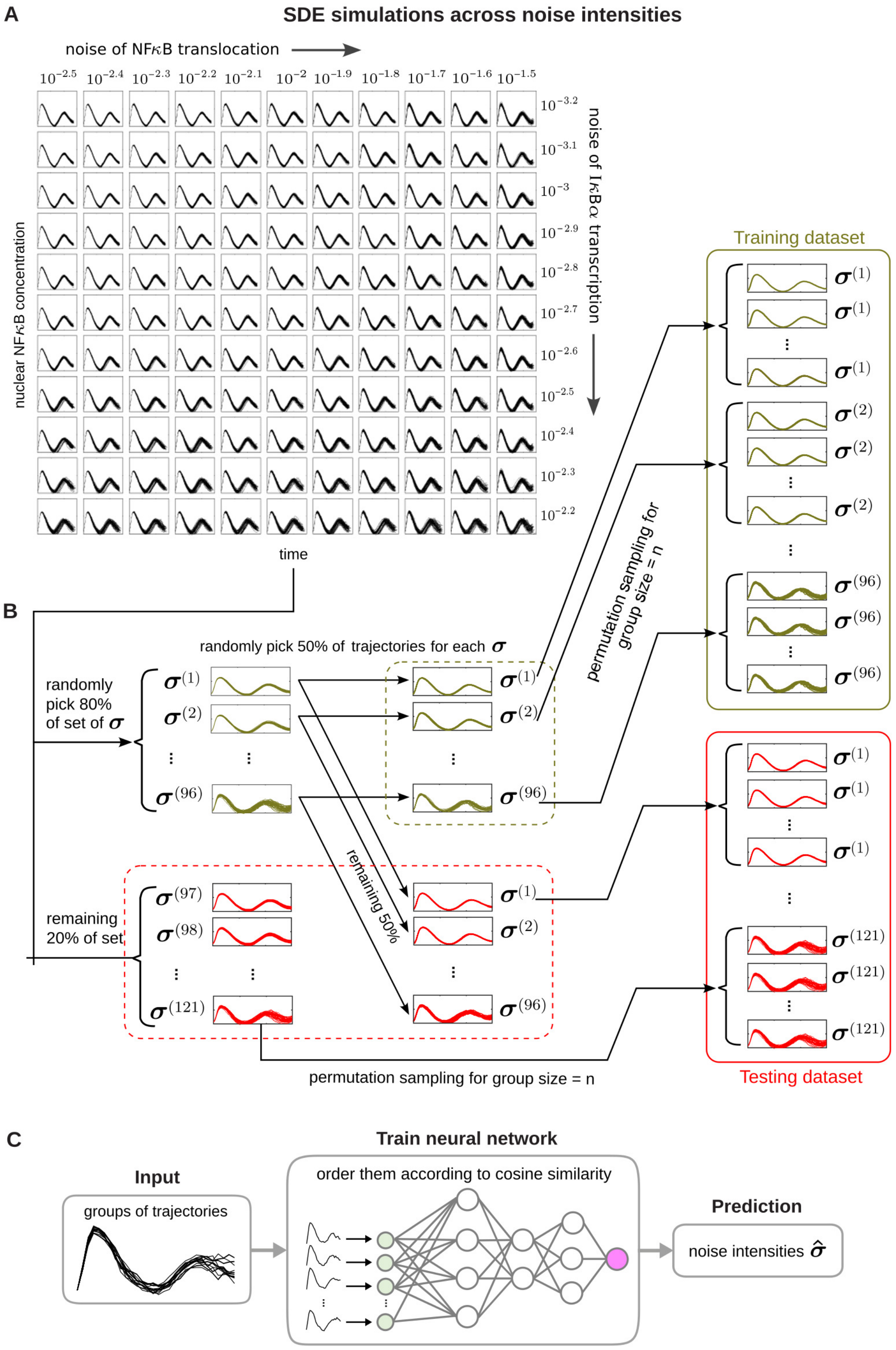

Figure E: **Workflow of using neural network to infer noise intensities from a group of trajectories.** A. NFκB SDE simulations under 121 different noise intensity settings. B. Schematic workflow for splitting the dataset into training and testing sets, where each group of trajectories is used to train and test the neural network for noise prediction. C. Schematic of the training of a neural network to predict noise intensities for each group of trajectories.

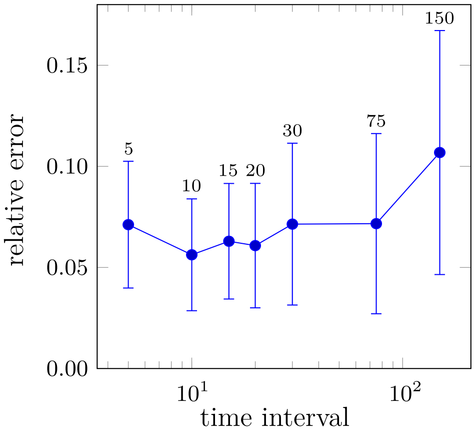

Figure F: **Neural network performance in inferring noise intensities from downsampled trajectories.** Relative error and uncertainty of inferred noise as a function of the trajectory sampling interval. Experimental trajectories were originally measured every 5 minutes over a 150-minute period; these measurements were then divided into trajectory groups for noise-intensity inference (Fig 8, Step 2). To assess the effect of longer intervals, the same dataset was downsampled to 10, 15, 20, 30, 75, and 150 minute sampling intervals.
