# Supplementary material for: Reconstructing noisy gene regulation dynamics using extrinsic-noise-driven neural stochastic differential equations
Source: PLoS Comput Biol. 2025 Sep 17;21(9):e1013462. doi: 10.1371/journal.pcbi.1013462 (PMC12513633; doi:10.1371/journal.pcbi.1013462)
Supplement: Attachment — Submitted filename: response1.pdf [file pcbi.1013462.s002.pdf]

PLoS Computational Biology

Dear Reviewer:

Thank you for your detailed comments and suggestions. We are grateful for your careful reading of our manuscript and have thoroughly revised it. Revisions of the manuscript are marked in blue, and below are our responses to your questions as well as a summary of the revisions we made based on your comments.

1.1 It's interesting to see that the authors can reconstruct temporal experimental data (Fig. 8). However, for a high-dimensional system (e.g., 52 dimensions referred in this work), how to analyze the stochastic dynamics is a paramount question, even if we already have a dynamical model or Neural SDE model, as the authors have emphasized the importance of external and intrinsic noise. For example, the energy landscape theory provides a way to study stochastic dynamics of high-dimensional biological systems (Kang and Li, Adv. Sci., 8: 2003133 (2021), Li and Wang, PNAS 111, 14130-14135 (2014)). So, I suggest the authors considering adding related discussions.

Response: We thank the reviewer for this insightful comment and valuable suggestion. We concur that analyzing high-dimensional stochastic dynamics represents a fundamental challenge in the field. The referenced works provide crucial theoretical frameworks for approximating the stochastic dynamics of high-dimensional biological systems. We have incorporated relevant discussions addressing this important aspect on page 20 of the revised manuscript. The modifications are outlined below:

- One way to address the high dimensionality problem is to make use of prior knowledge. Current examples in this manuscript did not utilize prior knowledge of the dynamics in the design of neural SDEs. We previously showed in [5] that the prior knowledge either in the form of ODEs or the self-consistent energy landscape can be used to facilitate the training of neural SDEs in a straightforward manner:

$$\mathrm{d}x = (F(x) + \mu_{\mathrm{NN}}(x))\,\mathrm{d}t + \sigma_{\mathrm{NN}}(x)\mathrm{d}W_t \quad (1)$$

  where $F(x)$ is the prior knowledge of the deterministic dynamics, *e.g.*, $F(x) = \nabla E(x)$m where $E(x)$ characterizes the energy potential landscape. Deviations from the prior knowledge as well as inferred intrinsic noise are encoded in neural networks $\mu_{\mathrm{NN}}(x)$ and $\sigma_{\mathrm{NN}}(x)$.

- In cases where only partial observation is available or when the dimension is too high, we can resort to approaches similar in [3, 2], including using proper methods to project the trajectories into lower dimensions and using methods similar to landscape to impose additional constraint on the neural networks μ_{NN} and σ_{NN} and reduce the underlying parameter space dimensions.

1.2 The authors have used Wasserstein distance. Can it be replaced by KL-divergence? If so, what is the advantage and disadvantage for using these two measures regarding current task?

Response: Thanks for your question. We have made a comparison between the Wasserstein distance and the KL divergence in our previous work [5]. Here, we provide a concise summary of the key findings. While both KL divergence and Wasserstein distance serve as measures for quantifying distributional differences, they differ fundamentally in their mathematical foundations. KL divergence operates purely on probability measures, whereas Wasserstein distance incorporates the metric structure of the underlying space. From a numerical perspective, the metric-based formulation demonstrates superior robustness compared to logarithmic computations. To illustrate this distinction, consider the following empirical distributions on $\mathbb{R}^1$: $A = \{1, 2\}$, $A' = \{0, 100\}$, and $B = \{1, 100\}$. Both $D_{\mathrm{KL}}(A\|B)$ and $D_{\mathrm{KL}}(A'\|B)$ are undefined, yet intuitively A exhibits greater dissimilarity to B than A' does. This is accurately captured by the Wasserstein distance:

$W_2(A, B) \approx 70 \gg 0.7 \approx W_2(A', B)$. We also added a reference that shows the Wasserstein distance is more suitable than the KL divergence for data in high-dimensional manifolds [1].

1.3 For reconstructing experimental data (Fig. 8), how many time points are there from experiments? And how does the proposed approach depend on the number (and resolution) of input experimental data point? Please elaborate this point.

Response: There are 31 time points (including 0) in the NFκB experimental data. As an additional sensitivity test, we also test how the prediction accuracy of intrinsic noise intensity depends on the number of observed time points. The results are shown in Fig. S5(D), and we found that the intensity noise inference accuracy is not very sensitive to the number of time steps within the range of this numerical experiment.

On the other hand, the Wasserstein-distance-based SDE reconstruction, which our END-nSDE is built upon, indeed yields a more accurate reconstructed neural SDE if a larger number of training trajectories is available, which is empirically shown in [4, 5]. However, in biological experiments, obtaining more training trajectories could also be more expensive. We have added a sentence in Conclusion to state that it is an interesting future direction to find out the number of training trajectories that can be practically obtained in real experiments and is necessary for an accurate reconstruction of the SDE using our END-nSDE approach.

References

[1] Martin Arjovsky, Soumith Chintala, and Léon Bottou. Wasserstein GAN, Jan 2017.

[2] Xin Kang and Chunhe Li. A Dimension reduction approach for energy landscape: identifying intermediate states in metabolism-EMT network. *Advanced Science*, 8(10), March 2021.

[3] Chunhe Li and Jin Wang. Landscape and flux reveal a new global view and physical quantification of mammalian cell cycle. *Proceedings of the National Academy of Sciences*, 111(39):14130–14135, September 2014.

[4] Mingtao Xia, Xiangting Li, Qijing Shen, and Tom Chou. An efficient Wasserstein-distance approach for reconstructing jump-diffusion processes using parameterized neural networks. *Machine Learning: Science and Technology*, 5:045052, 2024.

[5] Mingtao Xia, Xiangting Li, Qijing Shen, and Tom Chou. Squared Wasserstein-2 distance for efficient reconstruction of stochastic differential equations, 2024.
